# Rapid Analysis of Seven Polyamines in *Nephotettix cincticeps* by Using Ultra-Performance Liquid Chromatography-Triple Quadrupole Mass Spectrometry

**DOI:** 10.1155/2024/3302455

**Published:** 2024-04-16

**Authors:** Mingwen Zhang, Yunqiang Zhao, Zongwen Wang, Jintian Cheng

**Affiliations:** ^1^Fujian Provincial Key Lab of Coastal Basin Environment, Fujian Polytechnic Normal University, Fuqing 350300, China; ^2^Putian Public Security Bureau, Putian 351100, China; ^3^State Key Laboratory of Ecological Pest Control for Fujian and Taiwan Crops, College of Plant Protection and Key Lab of Biopesticide and Chemical Biology, Ministry of Education, Fujian Agriculture and Forestry University, Fuzhou, Fujian 350002, China

## Abstract

A fast, simple, and sensitive method for the simultaneous determination of seven polyamines in *Nephotettix cincticeps* was developed based on ultra-performance liquid chromatography-triple quadrupole mass spectrometry (UPLC-3Q-MS) together with liquid phase extraction. Polyamines in insect samples were extracted with HClO_4_ solution and then were separated and detected by UPLC-3Q-MS, which was equipped with a hydrophilic interaction liquid chromatography column, within 5 min without any derivatization procedure. The method has been successfully used to detect 7 polyamines in healthy and difluormethylornithine-treated adults of *Nephotettix cincticeps* with a method limit of detection and the method limit of quantitation of 24–139 pg/mg and 82–464 pg/mg, respectively, an intraday and interday relative standard deviation (RSD, *n* = 5) of 1.97–6.00% and 2.08–5.92% respectively, and a recovery of 86–115%. The success of this study provided a reliable method for the rapid and high-throughput detection of polyamines in the insect sample.

## 1. Introduction

Polyamines (PAs), such as spermine, putrescine, and spermidine, are aliphatic cations with a low molecular weight that contain two or more amino groups [1]. They are crucial for the growth and development in prokaryotes and eukaryotes [2]. As prominent polycations, PAs can interact with negatively charged molecules in the cell such as DNA, RNA, adenosine triphosphate, and phospholipids [3]. PAs were reported to implicate in diverse cellular processes such as protein synthesis [4], signal transduction [1], gene regulation [5], modulation of ion channel action [6], and cell proliferation [7].

PAs play important roles in the growth of the insect, such as embryonic development and vitellogenin synthesis [8], growth and metamorphosis [9], hormone metabolism [10], and behavior [11]. The concentration of PAs in insects was reported to change with the various developmental stages [12, 13]. PAs also were reported to affect the expression of olfactory-related genes in the diamondback moth [14] and modulate the phosphorylation of several antennary proteins of *Periplaneta americana* [15]. Since PAs are essential for many physiological and developmental processes in insects, detection of polyamine levels in insects can help to investigate PAs influences on insect development and the regulatory mechanisms of biotic and abiotic factors on insect polyamine biosynthesis pathways. However, few analytical methods were reported for the simultaneous detection of PAs in insects. Consequently, it is urgent to develop a simple, rapid, and sensitive method for simultaneously monitoring of PA levels in insects.

Various methods have been developed for the separation of PAs, such as thin-layer chromatography (TLC) [16], capillary electrophoresis (CE) [17], gas chromatography (GC) [18, 19], high-performance liquid chromatography (HPLC) [20], and ultra-performance liquid chromatography (UPLC) [21]. These separation methods usually coupled with several detectors, such as UV/Vis detector [22], fluorescence detector [23], electrochemical detector [24], and mass detector [25], to perform PAs analysis. Since natural PAs do not contain chromophore and fluorophore moieties, most analytical methods reported for PAs analysis require a derivatization procedure with the following different derivatization reagents: naphthalene-2, 3-dicarboxaldehyde [26] and o-phthalaldehyde [27] with fluorescence detection, dansyl chloride [28] and 2-naphthyloxycarbonyl chloride [29] with UV detection, and ferrocene derivatives [24] with electrochemical detection. In addition, precolumn derivatization of PAs, which produces nor-polar derivative, has been used in reversed-phase HPLC to enhance the separation resolution. However, chemical derivatization of PAs has some disadvantages such as long derivatization time, relatively complex procedure, and loss of accuracy. Recently, ultra-performance liquid chromatography-triple quadrupole mass spectrometry (UPLC-3Q-MS), which is one of the most powerful analytical techniques due to its high selectivity and sensitivity, good chromatographic resolution, short run time, and great antijamming capability, has been reported as a new method for analysis of biogenic amine in food [30]. In fact, UPLC-3Q-MS can perform the determination of PAs without a derivatization procedure in complicated matrices.

In this study, a simple, rapid, and sensitive method based on UPLC-3Q-MS together with a simple extraction procedure was developed for the detection of seven PAs in *Nephotettix cincticeps* (*N. cincticeps*), which is one of the most vital insect pests of rice as a rice plant pathogens' vector [31]. The method employs a hydrophilic interaction liquid chromatography (HILIC) column for the separation of the PAs without any requirement of derivatization. Two product ions (*m/z*) for the identification and quantification of the PAs were performed to obtain higher confidence in the results. The developed method can be applied for detecting the seven PA levels in *N. cincticeps* within 5 min. The success of this study provides a reliable method for the detection of PAs in insect or insect tissue.

## 2. Materials and Methods

### 2.1. Chemicals and Reagents

Cadaverine (Cad), 1,3-diaminopropane (Dap), 3, 3′-diaminodipropylamine (Dad), difluormethylornithine (DFMO), acetonitrile, and ammonium formate were purchased from Aladdin Chemistry Co., Ltd. (Shanghai, China). Putrescine (Put), spermine (Spm), and spermidine (Spd) were purchased as their hydrochloride salts from Sigma-Aldrich Co. (St. Louis, USA). Agmatine (Agm) was purchased as its sulfate salt from J & K Chemicals (Beijing, China). Perchloric acid (HClO_4_) and hydrochloric acid (HCl) were purchased from Sinopharm Chemical Reagent Co., Ltd. (Shanghai, China). All PAs were dissolved in 10 mM HCl solution to obtain 100 *μ*M of individual standard stock solutions and kept at −20°C. The standard mixture was gradient diluted from stocks by deionizing and stored at 4°C for up to 3 days. All standard samples and insect samples were stored in polypropylene tubes. The water used in the whole experiment was deionized water prepared by the Milli-Q equipment (Kerton lab Mini-D, UK).

### 2.2. *Nephotettix cincticeps*

*N. cincticeps* were incubated and reared in a cage (25 ± 1°C, 60–70% r. h.) and fed with rice plant. For the determination of PA levels in the healthy adults of *N. cincticeps*, 20 males were directly collected from the cage for polyamine extraction. For the determination of PA levels in *N. cincticeps* treated with DFMO, an enzyme-activated irreversible inhibitor of ornithine decarboxylase (ODC) [32], three groups, each consisting of 20 males, were supplied with different concentrations of DFMO (0, 10, and 20 mM) dissolved in 10% sucrose solution for 20 hours. Then, all the males were collected for polyamine extraction.

### 2.3. UPLC-3Q-MS System

The UPLC-3Q-MS system consists of an Agilent 1290 UPLC system and an Agilent 6460 triple quadrupole mass spectrometer (Agilent Technologies, USA). An Agilent ZORBAX HILIC Plus column (50 × 2.1 mm, 1.8 *μ*m particle size) was employed to achieve the separation of the PAs at a flow rate of 0.5 mL/min. The mobile phase consisted of acetonitrile solution (solvent A) and 90 mM ammonium formate solution (solvent B). The gradient elution program was as follows: (1) 0–2.50 min 30% solvent B, (2) 2.50–4.00 min from 30% to 50% solvent B, and (3) 4.00–5.00 min 80% solvent B. Total run time was 5 min and postrun time was 1 min. The injection volume was 2 *μ*L. The column temperature was set at 35°C. When the experiment was over, the HILIC column was flushed by activating buffer (0.1% formic acid/acetonitrile solution, 6 : 4 v/v) for 10 min and stored in acetonitrile/water solution (6 : 4 v/v).

The optimized conditions of MS/MS for the determination of PAs were as follows: drying gas temperature 350°C, gas flow 10 L/min, nebulizer 50 psi, capillary voltage 4000 V in the positive ionization mode, cell accelerator 4 V, and dwell time 50 ms. The optimized multiple reaction monitoring (MRM) conditions for PAs are listed in Supporting Information Table S1.

### 2.4. Determination of Polyamines in *Nephotettix cincticeps* Samples

First, collected *N. cincticeps* were snap-frozen in liquid nitrogen and each adult was weighed (about 2 mg) by electronic balance (Ohaus Co., Pine Brook, NJ, USA) with a maximum permissible error of ±0.01 mg. The weighed *N. cincticeps* samples were homogenized in ice-cold HClO_4_ (0.8 M) with a ratio of 20 *μ*L/mg (volume/mass ratio) by G50 motor-driven tissue grinder with plastic grinder head (Sangon Biotech, China) for 1 min, followed by centrifuging at 16162 × g for 4 min by Sigma 1–14 microcentrifuge (Sigma Corp., Germany). Then, the extract solution was collected. The organizational residue was repeatedly extracted once again in the same manner. Finally, two extracts were mixed and diluted 60-fold with deionized water. The seven PAs in the final solution were separated and detected by UPLC-3Q-MS under optimal conditions. The samples spiked with seven PAs were also detected in the same manner to obtain recovery.

### 2.5. Validation and Statistics

The analytical method was fully validated by evaluating specificity, linearity range, intra- and interday precision and accuracy, limits of detection (LODs), limits of quantitation (LOQs), method limit of detection (MLOD), the method limit of quantitation (MLOQ), and recovery. All the data were expressed as means ± standard deviations (SDs) and significance was determined at *p* < 0.05 by analysis of variance (ANOVA) followed by Tukey's test.

## 3. Results and Discussion

### 3.1. Optimization of the MS Conditions

Under the ESI positive mode, characterization of the mass spectra was performed by direct injection of a standard solution of each PA (10 *μ*M). The precursor ion of each PA corresponds to the protonated molecule ion [M + H]^+^. Then, the fragmentor voltage was optimized to obtain the highest intensity of the precursor ion. In the product ion spectra, Cad, Put, and Dap lost one ammonia molecule as a result to produce [M + H − NH_3_]^+^ fragment and Spd lost two ammonia molecules to produce [M + H − 2NH_3_]^+^ fragment. Spm and Agm lost a large fragment to produce [M + H − (CH_2_)_3_N_2_H_4_ − NH_3_]^+^ fragment (*m/z* 112) and [M + H − CN_3_H_5_]^+^ fragment (*m/z* 72), respectively. According to the European Commission Decision 2002/657/EC, two main characteristic fragment ions should be selected as the qualitative and quantitative ions and the collision voltage should be optimized to achieve the maximum intensity of the characteristic fragment ions. The characteristic ion pairs, fragmentor, and collision conditions of seven target PAs were optimized and presented in Table S1 (see supporting information, SI).

### 3.2. Chromatography

The recommended separation mechanism of the HILIC column is based on the differential distribution of the injected analyte between the acetonitrile-rich mobile phase and a water-enriched layer adsorbed onto the hydrophilic stationary phase [33]. The chromatographic conditions including gradient elution program, concentration of buffering salt, pH of buffer, and temperature were optimized.

**Figure 1:**
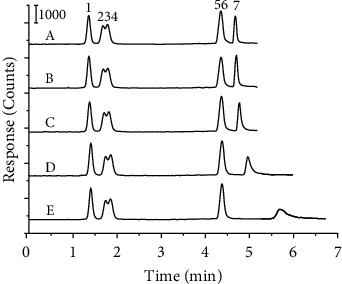
MRM chromatogram of Spm under different percentages of solvent B in the third step of the gradient elution program. (A) 90% solvent B, (B) 80% solvent B, (C) 70% solvent B, (D) 60% solvent B, (E) 50% solvent B, (1) Agm, (2) Cad, (3) Dap, (4) Put, (5) Dad, (6) Spd, (7) Spm.

The gradient elution program was optimized first. Increasing the concentration of the organic solvent increases the thickness of the water-enriched layer on the stationary phase surface, which leads to an increased retention time of the polar analyte. In this study, Spm, which is the strongest polar compound of the seven PAs, is the hardest to elute from the HILIC column. So, the percentage of solvent B (90 mM ammonium formate solution) in the third step of the gradient elution program, which was employed to elute Spm, was optimized from 50% to 90%. As shown in Figure 1, when the percentage of solvent B increased, the elution of Spm became easy and the peak width of Spm became narrowed and symmetrical. Considering the peak height and shape of Spm, 80% solvent B was selected as the optimum condition.

The concentration of buffering salts in the mobile phase affects the signal of analytes and the electrostatic interactions between analytes and the stationary phase. Increasing the concentration of buffering salts will decrease the retention time of polar analytes as a result of decreasing electrostatic interactions. On the other hand, a high concentration of buffering salts favors the elution of the polar compounds and provides a suitable sensitivity. However, high concentrations of buffering salts may lead to ion suppression phenomena, which tend to inhibit the signal of analytes. As shown in Figure S1 (see SI), when the concentration of ammonium formate solution increased from 70 mM to 110 mM, the retention time of all PAs decreased, and the signals of Agm, Dad, Spd, and Spm increased while the signals of other three PAs decreased. Considering all the signals of PAs, a 90 mM ammonium formate solution was selected.

The mobile phase pH affects the hydrophilicity of the analyte and the interaction between the analytes and the stationary phase. A lower pH value provides a higher concentration of hydrogen proton which facilitates the elution of the analytes from the stationary phase. Moreover, a high concentration of hydrogen protons can contribute to the ionization of the amino groups of PAs, thereby increasing their signal value. Conversely, an excessive concentration of hydrogen protons can lead to ion suppression, resulting in a decrease in the signal value of PAs. As shown in Figure S2 (see SI), an increase in the pH of the ammonium formate solution from 3.5 to 5.5 resulted in an increase in the retention time for all PAs. In addition, the signals for Spm, Spd, and Dad decreased, while the signals for the other four PAs increased. Considering all the signals of PAs, the optimal pH of ammonium formate solution was selected as 4.5.

Column temperature can affect analyte diffusivity, mobile phase viscosity, and analyte-transferring enthalpy between mobile and stationary phases. Limited by the operating temperature of ZORBAX HILIC Plus column (≤40°C), the range of column temperature from 25°C to 40°C was investigated. From the results shown in Figure S3 (see SI), when the column temperature increased, the retention time of all PAs had a tiny increase, and signals of PAs had a tiny increase or decrease except that of Put increased significantly. Considering the column lifetime and the signal of PAs, the optimal column temperature was selected as 35°C.

**Figure 2:**
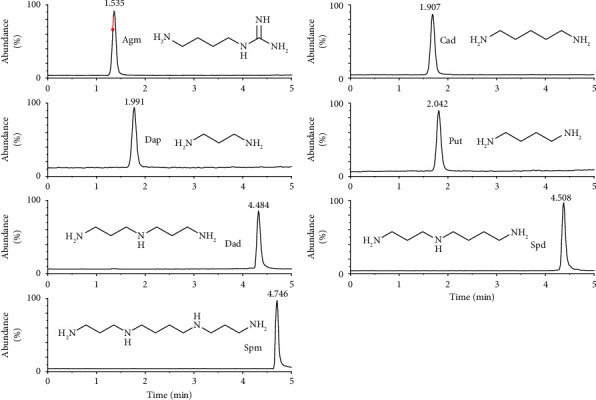
MRM chromatogram of the mixed standard solution.

Under the optimized UPLC-3Q-MS conditions, seven PAs were detected in 5 min with sharp symmetrical peak shapes and four important PAs (Agm, Cad, Spd, and Spm) formed from the arginase/ornithine decarboxylase pathway achieved baseline separation (Figure 2). The chromatographic run time was faster than most methods designed to simultaneously determine PAs [34, 35].

### 3.3. Optimization for the Extraction of Polyamines in *Nephotettix cincticeps*

**Figure 3:**
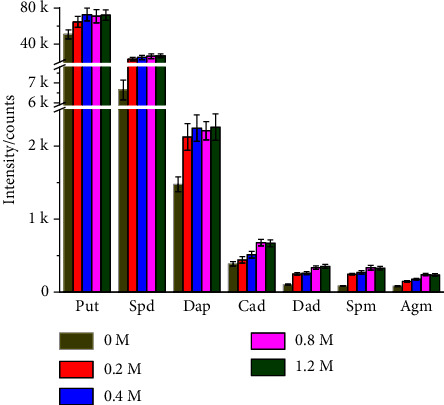
The effects of different concentrations of HClO_4_ on the signals of seven PAs (*n* = 5). Data are expressed as the mean (SD).

In order to obtain a rapid and simple method for analyzing PAs in insects, a traditional HClO_4_ extract method without solid phase extraction and derivatization procedure was adopted [36]. The effect of HClO_4_ concentration on the extraction efficiency was investigated in the range from 0 to 1.2 M. As shown in Figure 3, for most of the polyamine, the signals (peak area) were increased with increasing HClO_4_ concentration and reached the platform at 0.8 M HClO_4_. Hence, 0.8 M HClO_4_ was selected as the optimal HClO_4_ concentration. Then, extraction cycles were optimal with 0.8 M HClO_4_. The solution of each extraction was diluted and detected. In the third extraction, all of the PAs were undetected except that Put was detected with negligible signal (data not shown). Therefore, two extraction cycles were selected. Compared to the derivatization procedure which needed a long derivatization time (>1.5 h) and cumbersome experimental step [28], the HClO_4_ extract method is faster (about 10 min) and simpler.

### 3.4. Method Validation

Linearity was tested in 3 days at six concentration levels. As shown in Table 1, the linear correlation coefficients between the peak area and concentrations were not less than 0.9990 for all the PAs. The instrument detection limits (LODs) (3 *σ*/S, the concentration necessary to yield a net signal equal to three times the SD of the background) and limits of quantification (LOQs) (10 *σ*/S) were calculated in the ranges of 10.38–58.64 pg/g and 34.10–194.13 pg/g, respectively. Compared to other MS-based methods for the detection of PAs, the developed method not only achieves comparable detection limits and linear ranges but also provides a shorter analysis time (see Table S2). The method limits of detection and quantitation are also shown in Table 1. Then, considering the dilution ratio of the sample and the volume/mass ratio of extraction, the method limit of detection (MLOD) and the method limit of quantitation (MLOQ) were calculated in the ranges of 24–139 pg/mg and 82–464 pg/mg, respectively.

Recoveries were determined by analyses of sample spiked with corresponding concentrations of each polyamine. Five replicates were performed at each spiked level. The results of recovery experiments are shown in Table 2. The recovery rates of spiked samples ranged from 86% to 115%, with an intraday relative standard deviation (RSD %) ranging from 1.97% to 6.00% and an interday RSD % ranging from 2.08% to 5.92%. These results highlight the precision and reliability of the method in analyzing the samples.

### 3.5. Determination of Polyamines in *Nephotettix cincticeps*

**Figure 4:**
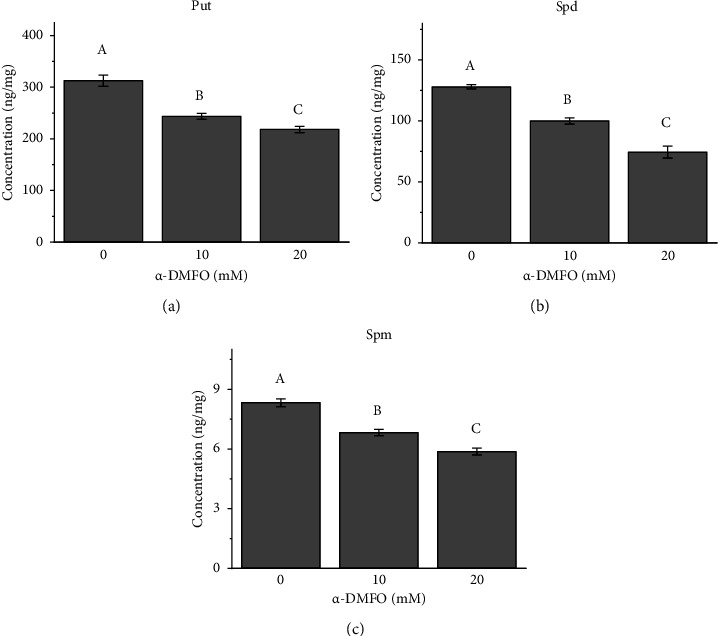
The effect of DFMO on Put (a), Spd (b), and Spm (c) in *N. cincticeps*. Each histography bar represents the mean (SD). ^A–C^Different letters indicate significant differences (*p* < 0.05) by analysis of variance (ANOVA) and Tukey's test.

The optimized and validated UPLC-3Q-MS method was applied for the analysis of PAs in the adult of *N. cincticeps*. The MRM chromatogram of PAs in *N. cincticeps* is shown in Figure S4, and the concentration of PAs in the *N. cincticeps* is presented in Table 2. Seven PAs were detected with concentrations in the range from 1.27 ng/mg for Agm to 314.12 ng/mg for Put. Furthermore, the concentration of PAs in adults of *N. cincticeps* treated with different concentrations of DFMO (0, 10, and 20 mM) was tested. DFMO is an enzyme-activated irreversible inhibitor of ODC, which is the first enzyme in the leading pathway to the biosynthesis of the Put, Spd, and Spm in cells [32]. As shown in Figure 4, the concentration of Put, Spd, and Spm decreased significantly by the increased concentration of DFMO. On the other hand, the concentration of the other PAs between the groups fed with and without DFMO did not show significant differences (see Table S3). These results demonstrated the developed method is capable of determining a change in the PA levels in insects affected by external factors.

## 4. Conclusions

In summary, a rapid, simple, and sensitive analytical method based on UPLC-3Q-MS equipped with a HILIC column was developed for the simultaneous detection of seven PAs in *N. cincticeps*. The HILIC column was employed to directly separate underivatized PAs, which were easily extracted by HCLO_4_ without the derivatization procedure and with a satisfactory recovery from 86% to 115%. Under the optimized UPLC-3Q-MS conditions, the developed method was validated showing satisfactory analytical performance. Seven PAs were detected with sharp symmetrical peak shapes in 5 min, and the total time for individual sample preparation, separation, and detection was about 15 min. With the help of developed methods, the concentrations of seven PAs in the adult of *N. cincticeps* were detected in the range from 1.27 ng/mg for Agm to 314.12 ng/mg for Put. The method was successfully applied to investigate the effect of DFMO on the PA levels in *N. cincticeps*. The success of this study provided a rapid and reliable approach for detecting PAs in insect or insect tissue and investigating the external factors that influence the PA level in insects.

## Figures and Tables

**Table 1:** Linear ranges, calibration curves, correlation coefficient (*R*^2^), limits of detection and quantitation (LODs and LOQs), and method limits of detection and quantitation (MLODs and MLOQs).

PAs	Linear ranges (pg/mg)	Calibration curves	*R* ^2^	LODs (pg/g)	LOQs (pg/g)	MLODs (pg/mg)	MLOQs (pg/mg)
Dap	0.074–37	*y* = 28.87*x* + 19.07	0.9999	10.38	34.10	24	82
Put	0.088–176.3	*y* = 55.80*x* + 1048	0.9990	14.10	46.72	33	111
Cad	0.102–51.1	*y* = 76.79*x* − 3.630	0.9999	14.30	47.00	34	114
Agm	0.13–65	*y* = 83.11*x* + 24.17	0.9999	16.90	57.20	42	139
Dad	0.131–65.6	*y* = 43.35*x* − 29.09	0.9997	28.86	98.4	71	235
Spd	0.145–145.2	*y* = 75.09*x* + 201.8	0.9999	15.97	52.27	37	125
Spm	0.202–101.1	*y* = 40.06*x* + 22.58	0.9995	58.64	194.13	139	464

**Table 2:** Concentration of PAs in *N. cincticeps* given in ng/mg ± standard deviation, spiked concentration (ng/mg), mean recoveries, and inter- and intraday precision.

PAs	Concentration in *N. cincticeps* (ng/mg)^*∗*^	Spiked concentration (ng/mg)	Recovery (%)	Intraday RSD % (*n* = 5)	Interday RSD % (*n* = 5)
Dap	23.76 ± 2.98	25	92	2.04	2.08
		50	93	3.43	3.47
		100	94	4.94	5.32

Put	314.12 ± 39.08	100	96	2.49	2.79
		200	94	3.55	3.64
		400	93	2.93	3.31

Cad	2.25 ± 0.39	5	97	2.39	3.21
		10	96	2.98	3.72
		500	86	1.97	2.90

Agm	1.27 ± 0.10	6	101	6.00	5.33
		12	99	2.46	3.24
		60	98	4.31	4.59

Dad	3.16 ± 0.41	6	115	4.13	4.18
		12	110	5.76	5.92
		60	101	4.38	5.17

Spd	126.88 ± 8.56	120	95	4.33	5.51
		240	103	3.17	4.26
		480	100	2.88	3.07

Spm	8.06 ± 0.70	10	95	2.85	2.92
		20	98	4.74	4.90
		100	99	3.52	4.12

^*∗*^Data are expressed as the mean ± SD.

## Data Availability

The data used to support the findings of this study are included within the supplementary information file.

## Supplementary Materials

Supplementary Materials The HPLC-MS/MS conditions used for the determination of PAs are detailed in the supplementary materials. Table S1: multiple reaction monitoring (MRM) conditions optimized for PAs. Table S2: literature values of limits of detection (LODs), linear ranges, and correlation coefficient for the determination of polyamines. Table S3: the concentration of seven PAs in the *N. cincticeps* fed with different concentrations of *α*-DMFO. Figure S1: the retention time and peak area of polyamines under different concentrations of ammonium formate solution. Figure S2: the retention time and peak area of polyamines under different pH of ammonium formate solution. Figure S3: the retention time and peak area of polyamines under different column temperatures. The data of Figure S1–S3 were obtained by determining a 100 nM standard mixture. PA concentrations: Spm: 20.2 ng/mL, Spd: 14.5 ng/mL, Dad: 13.1 ng/mL, Put: 8.8 ng/mL, Dap: 7.4 ng/mL, Cad: 10.2 ng/mL, Agm: 13.0 ng/mL). Figure S4: MRM chromatogram of PAs in *N. cincticeps*.
