# Supplementary material for: Rapid Analysis of Seven Polyamines in Nephotettix cincticeps by Using Ultra-Performance Liquid Chromatography-Triple Quadrupole Mass Spectrometry
Source: J Anal Methods Chem. 2024 Apr 16;2024:3302455. doi: 10.1155/2024/3302455 (PMC11178422; doi:10.1155/2024/3302455)
Supplement: Supplementary Materials — The HPLC-MS/MS conditions used for the determination of PAs are detailed in the supplementary materials. Table S1: multiple reaction monitoring (MRM) conditions optimized for PAs. Table S2: literature values of limits of detection (LODs), linear ranges, and correlation coefficient for the determination of polyamines. Table S3: the concentration of seven PAs in the N. cincticeps fed with different concentrations of α-DMFO. Figure S1: the retention time and peak area of polyamines under different concentrations of ammonium formate solution. Figure S2: the retention time and peak area of polyamines under different pH of ammonium formate solution. Figure S3: the retention time and peak area of polyamines under different column temperatures. The data of Figure S1–S3 were obtained by determining a 100 nM standard mixture. PA concentrations: Spm: 20.2 ng/mL, Spd: 14.5 ng/mL, Dad: 13.1 ng/mL, Put: 8.8 ng/mL, Dap: 7.4 ng/mL, Cad: 10.2 ng/mL, Agm: 13.0 ng/mL). Figure S4: MRM chromatogram of PAs in N. cincticeps. [file 3302455.f1.docx]

**Rapid analysis of** **seven polyamines in** ***Nephotettix cincticeps* by** **ultra-performance liquid chromatography-****triple quadrupole mass spectrometry**

Mingwen Zhang,^1,*^ Yunqiang Zhao,^2^ Zongwen Wang,^3^ Jintian Cheng ^1,*^

^1^ Fujian Provincial Key Lab of Coastal Basin Environment, Fujian Polytechnic Normal University, Fuqing 350300, China

^2^ Putian Public Security Bureau, Putian 351100, China

^3^ State Key Laboratory of Ecological Pest Control for Fujian and Taiwan Crops, College of Plant Protection & Key Lab of Biopesticide and Chemical Biology, Ministry of Education, Fujian Agriculture and Forestry University, Fuzhou, Fujian 350002, China

Correspondence should be addressed to: Mingwen Zhang, [zhangmw@stu.fpnu.edu.cn,](mailto:zhangmw@stu.fpnu.edu.cn;) [mwzhang1989@163.com](mailto:zhangmw@fpnu.edu.cn;); Jintian Cheng, 184835975@qq.com

**1. The HPLC-MS/MS conditions used for the determination of PAs**

The optimized MS/MS conditions for the determination of PAs are as follows: drying gas temperature 350 °C, gas flow 10 L/min, nebulizer 50 psi, capillary voltage 4000 V in positive ionization mode, cell accelerator 4 V, and dwell time 50 ms.

**TABLE S1** Multiple Reaction Monitoring (MRM) conditions optimized for PAs.

| PAs | Precursor Ion  [M + H]^+^ | Product Ion 1 (*m/z*) | Product Ion 2 (*m/z*) | Fragmentor (V) | Collision Energy (V) |
| --- | --- | --- | --- | --- | --- |
|  |  |  |  |  |  |
|  |  |  |  |  |  |
| Dap | 75 | 58 | 30 | 40 | 8/17 |
| Put | 89 | 72 | 30 | 40 | 7/19 |
| Cad | 103 | 86 | 41 | 65 | 8/25 |
| Agm | 131 | 72 | 60 | 70 | 14/9 |
| Dad | 132 | 98 | 58 | 80 | 14/19 |
| Spd | 146 | 72 | 112 | 80 | 16/13 |
| Spm | 203 | 112 | 129 | 75 | 16/11 |

1. **(B)**

**FIGURE S1** The retention time and peak area of polyamines under different concentrations of ammonium formate solution. PA concentrations: Spm 20.2 ng/mL, Spd 14.5 ng/mL, Dad 13.1 ng/mL, Put 8.8 ng/mL, Dap 7.4 ng/mL, Cad 10.2 ng/mL, Agm 13.0 ng/mL).

1. **(B)**

**FIGURE S2** The retention time and peak area of polyamines under different pH of ammonium formate solution. PA concentrations: Spm 20.2 ng/mL, Spd 14.5 ng/mL, Dad 13.1 ng/mL, Put 8.8 ng/mL, Dap 7.4 ng/mL, Cad 10.2 ng/mL, Agm 13.0 ng/mL).

1. **(B)**

**FIGURE S3** The retention time and peak area of polyamines under different column temperatures. PA concentrations: Spm 20.2 ng/mL, Spd 14.5 ng/mL, Dad 13.1 ng/mL, Put 8.8 ng/mL, Dap 7.4 ng/mL, Cad 10.2 ng/mL, Agm 13.0 ng/mL).

**TABLE S2** Literature values of limits of detection (LODs), Linear ranges, and correlation coefficient for determination of polyamines.

| Analytical methods | Polyamines | LODs | Linear ranges | Correlation coefficient (R^2^) | Analysis time | Sample |
| --- | --- | --- | --- | --- | --- | --- |
| HPLC-Q-TOF/MS^[a]^ | Cad | 0.02 ng/mL | 0.4-200.0 ng/mL | 0.9944 | ~ 20 min | human plasma |
|  | Dap | 0.1 ng/mL | 0.4-200.0 ng/mL | 0.9904 |  |  |
|  | Put | 0.1 ng/mL | 0.4-200.0 ng/mL | 0.9882 |  |  |
|  | Spm | 0.05 ng/mL | 0.4-200.0 ng/mL | 0.9874 |  |  |
|  | Spd | 0.1 ng/mL | 0.4-200.0 ng/mL | 0.9878 |  |  |
| GC-MS^[b]^ | Put | 1.0 ng/g | 1-1000 ng/mL | 0.994 | ~ 20 min | Postmortem brain cortex |
|  | Spm | 100 ng/g | 100-100000 ng/mL | 0.776 |  |  |
|  | Spd | 10 ng/g | 100-100000 ng/mL | 0.998 |  |  |
| GC-SIM-MS^[c]^ | Cad | 10 ng/g | 5-200 ng/mL | 0.998 | ~ 18 min | Human hair |
|  | Dap | 1 ng/g | 5-200 ng/mL | 0.968 |  |  |
|  | Put | 5 ng/g | 5-200 ng/mL | 0.958 |  |  |
|  | Spm | 5 ng/g | 10-1000 ng/mL | 0.994 |  |  |
|  | Agm | 10 ng/g | 10-1000 ng/mL | 0.998 |  |  |
| UPLC-MS/MS^[d]^ | Cad | 10.38 pg/g | 0.074-37 pg/mg | 0.9999 | ~ 5 min | N. cincticeps |
|  | Dap | 14.10 pg/g | 0.088-176.3 pg/mg | 0.9999 |  |  |
|  | Dad | 14.30 pg/g | 0.102-51.1 pg/mg | 0.9997 |  |  |
|  | Put | 16.90 pg/g | 0.13-65 pg/mg | 0.9990 |  |  |
|  | Spm | 28.86 pg/g | 0.131-65.6 pg/mg | 0.9995 |  |  |
|  | Spd | 15.97 pg/g | 0.145-145.2 pg/mg | 0.9999 |  |  |
|  | Agm | 58.64 pg/g | 0.202-101.1 pg/mg | 0.9999 |  |  |

[a] HPLC-Q-TOF/M method from Reference S1.

[b] GC-MS method from Reference S2.

[c] GC-SIM-MS method from Reference S3.

[d] UPLC-MS/MS method from this work.

**
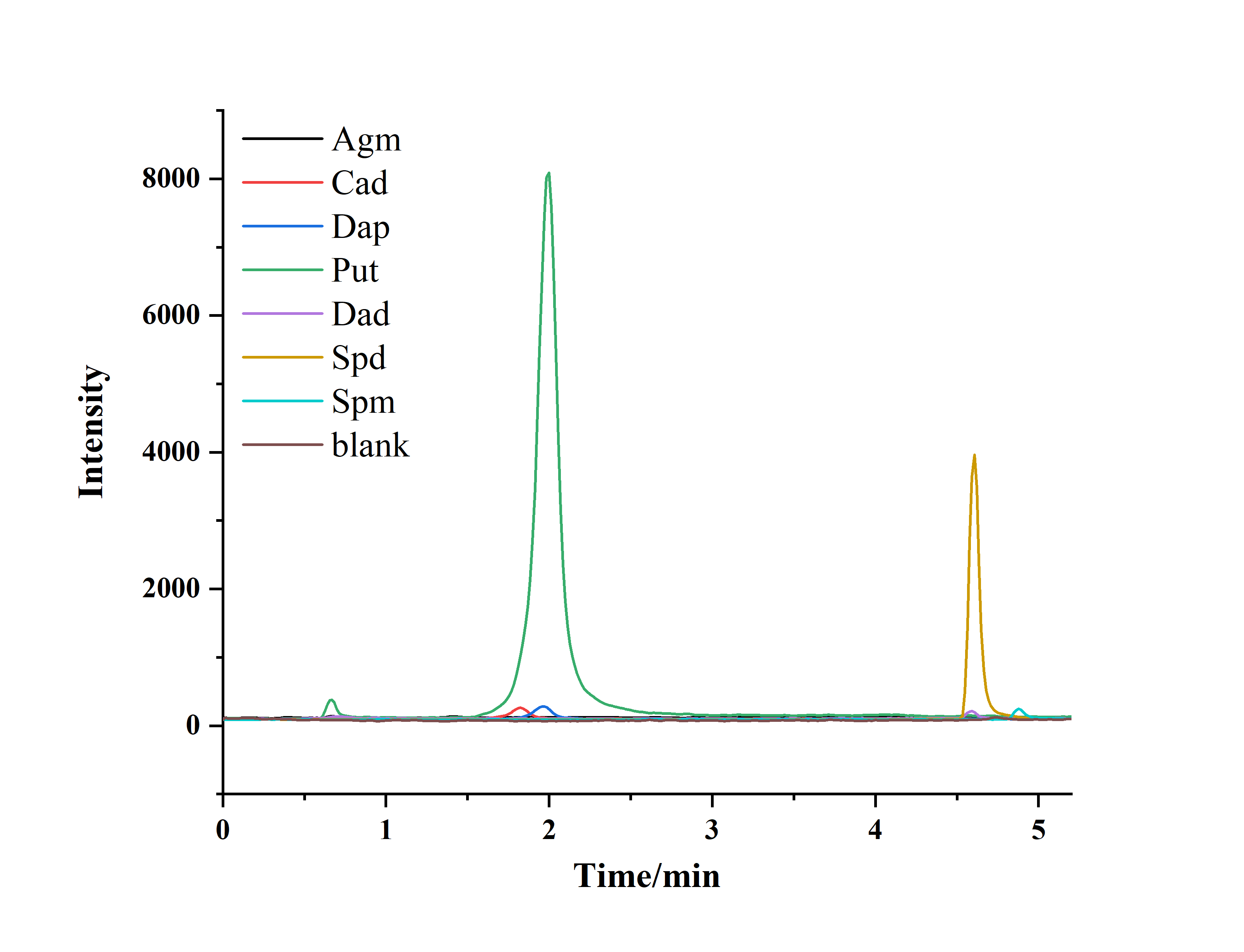
**

**FIGURE S4** MRM chromatogram of PAs in N. cincticeps.

**TABLE S3** The concentration of seven PAs in the *N. cincticeps* fed with different concentrations of α-DMFO.

| PAs | Group 1^a^  (ng/mg) | Group 2^b^  (ng/mg) | Group 3^c^  (ng/mg) |
| --- | --- | --- | --- |
| Dap | 21.20 ± 2.84 | 20.06 ± 2.81 | 20.39 ± 2.01 |
| Put | 312.70 ± 42.03^A^ | 243.69 ± 22.31^B^ | 218.21 ± 23.03^B^ |
| Cad | 2.14 ± 0.15 | 2.11 ± 0.34 | 2.24 ± 0.41 |
| Agm | 1.35 ± 0.09 | 1.29 ± 0.18 | 1.33 ± 0.20 |
| Dad | 3.16 ± 0.36 | 2.74 ± 0.45 | 3.07 ± 0.61 |
| Spd | 127.94 ± 7.03^A^ | 113.63 ± 9.70^B^ | 99.91 ± 18.74^C^ |
| Spm | 8.33 ± 0.76^A^ | 6.83 ± 0.60^B^ | 5.87 ± 0.67^C^ |

a: Groups of *N. cincticeps* feed with 10% sucrose solution.

b: Groups of *N. cincticeps* feed with 10 mM α-DMFO in 10% sucrose solution.

c: Groups of *N. cincticeps* feed with 20 mM α-DMFO in 10% sucrose solution.

Data are expressed as mean ± SD. ^A–C^ Different letters indicate significant differences (P < 0.05) by analysis of variance (ANOVA) and Tukey’s test.

References

S1. R. Liu, K. S. Bi, Y. Jia, Q. Wang, R. Yin, Q. Li, “Determination of polyamines in human plasma by high-performance liquid chromatography coupled with Q-TOF mass spectrometry”, Journal of Mass Spectrometry, Vol. 47, pp. 1341-1346, 2012.

S2. G. G. Chen, G. Turecki, O. A. Mamer, “A quantitative GC-MS method for three major polyamines in postmortem brain cortex”, Journal of Mass Spectrometry, Vol. 44, pp. 1203-1210, 2009.

S3. M. H. Choi, K. R. Kim, B. C. Chung, “Determination of hair polyamines as N-ethoxycarbonyl-Npentafluoropropionyl derivatives by gas hromatography–mass spectrometry”, Journal of Chromatography A, Vol. 897, pp. 295-305, 2000.
